# Exploring the causal relationship between gastroesophageal reflux and oral lesions: A mendelian randomization study

**DOI:** 10.3389/fgene.2022.1046989

**Published:** 2022-11-29

**Authors:** Linjing Shu, Xu Tong

**Affiliations:** ^1^ Stomatological Hospital of Chongqing Medical University, Chongqing, China; ^2^ Chongqing Key Laboratory of Oral Diseases and Biomedical Sciences, Chongqing Medical University, Chongqing, China; ^3^ Chongqing Municipal Key Laboratory of Oral Biomedical Engineering of Higher Education, Chongqing Medical University, Chongqing, China

**Keywords:** mendelian randomization, causal relationship, gastroesophageal reflux disease, oral disease, periodontitis, toothache, oral ulcer

## Abstract

**Background:** Clinical observations and retrospective studies have observed that patients with gastroesophageal reflux disease (GERD) have an increased probability of dental erosion, periodontitis and oral mucosal lesions and other common oral lesions. However, whether there is a genetic causal relationship between GERD and the occurrence of oral lesions has not been reported.

**Methods:** In this study, we extracted instrumental variables from the largest published summary statistics of the oral lesion phenotype GWAS in UK Biobank (UKBB) and GERD GWAS. Then, we performed a causal inference analysis between GERD and common oral lesions by mendelian randomization (MR) analysis with the R package “TwoSampleMR”.

**Results:** We observed a significant causal relationship between GERD and several common oral lesion phenotypes (painful gums, loose teeth, toothache, and mouth ulcers). GERD showed a positive correlation with the occurrence of these oral lesions. After removing outlier SNPs *via* the MR-PRESSO package, our conclusions were still robust.

**Conclusion:** Our findings provide the first evidence for a genetic causal effect of GERD on oral lesion pathogenesis. For patients with confirmed GERD, attention should be paid to taking interventions to prevent the occurrence of oral lesions.

## Introduction

As an important part of the digestive system, the occurrence of digestive system diseases can also lead to changes in the oral environment. Gastroesophageal reflux disease (GERD) is a series of chronic symptoms and esophageal mucosal damage caused by reflux of gastric contents due to dysfunction of the lower esophageal sphincter. The prevalence of GERD in adults in Western countries ranges from 10% to 20% (Chen et al., 2014; El-Serag et al., 2014). In addition to affecting the esophagus, GERD leads to a series of extraesophageal symptoms known as extraesophageal syndrome, including chronic cough, hoarseness, asthma, globus sensation, sleep disturbance, and oral lesions (Jung et al., 2020). Various forms of dental erosion are considered to be the most important oral manifestations of GERD, and the relationship between GERD and dental erosion and tooth loss has been widely observed. Picos et al. (2013) found that dental erosion prevalence in patients with GERD was 35% by investigating the oral health status of GERD patients. Similarly, due to the defect of tooth enamel, the incidence of dental caries in GERD patients also increased (Linnett et al., 2002). Furthermore, the colonization of the tooth surfaces by S. salivarius and Streptococcus mutans was significantly increased in children with GRED (Ersin et al., 2006). In addition, the relationship between changes in salivary flow rate and salivary buffering capacity (Hauk, 2018), changes in taste (Steele, 2016), damage to the oral mucosa, and the onset of chronic periodontitis (Song et al., 2014) have also been reported (Jajam et al., 2017). In previous studies, an increased incidence of tooth erosion, periodontitis, oral mucosal lesions, and dysgeusia was observed in patients with GERD. Although there appears to be a strong association between GERD and oral lesions, previous evidence from either cross-sectional or case-control studies, the direction of the causal relationship between oral lesions and GERD remains uncertain.

Mendelian randomization methods use genetic variation as an instrumental variable reflecting exposure factors (intermediate phenotypes). Since the alleles of genetic variation follow the Mendelian independent distribution law of random separation and combination from parents to offspring when gametes are formed, the random distribution of alleles makes the process of randomization of genetic variation in the population. The relationship between genetic variation and exposure is fixed during conception, independent of postnatal environmental exposures, confounding, and outcomes, and throughout the lifespan to rationalize causal timing (Lawlor et al., 2008). At the same time, genetic variation can be directly and accurately measured, and it can be used as an instrumental variable while avoiding the bias introduced by measurement error. Therefore, mendelian randomization takes genetic variation as an instrumental variable of the exposure factors to be studied, and it is feasible to use the relationship of “genetic variation-study outcome” to simulate the relationship of “exposure factor-study outcome” to infer disease etiology (Neeland and Kozlitina, 2017). In this study, we performed a causal inference analysis between GERD and common oral lesions by mendelian randomization analysis.

## Methods

### Instrumental variable selection

The GWAS summary data for gastroesophageal reflux comes from the largest published GWAS study of gastroesophageal reflux in European populations (Ong et al., 2022), which included a total sample size of 602,604, including 129,080 cases and 473,524 controls. First, this study selected SNPs that reached the genome-wide significant threshold (*p* < 5 × 10^−8^). At the same time, in order to avoid potential bias caused by linkage disequilibrium (LD) relationship between SNPs, we set the physical distance between SNPs >10 000 kb by setting the clump_data function in the TwoSampleMR package, and the R^2^ of LD between genes <0.001, the instrumental variable is finally obtained.

GWAS summary statistics data for several common oral disease phenotypes, including loose teeth, bleeding gums, toothache, and oral ulcers were downloaded from UKBB, and β, SE, and p values were extracted for these outcome factors.

The published data used in this study were derived from analyses limited to European population data, and basic information on these subjects is presented in Table 1.

**TABLE 1:** Basic information on the GWAS applied in this study.

GWAS ID	Year	Trait	Consortium	Sample size	Number of SNPs
ebi-a-GCST90000514	2021	Gastroesophageal reflux disease	NA	6, 02, 604	23, 20, 781
ukb-b-11161	2018	Mouth/teeth dental problems: Painful gums	MRC-IEU	4, 61, 113	98, 51, 867
ukb-b-12849	2018	Mouth/teeth dental problems: Loose teeth	MRC-IEU	4, 61, 113	98, 51, 867
ukb-b-19191	2018	Mouth/teeth dental problems: Toothache	MRC-IEU	4, 61, 113	98, 51, 867
ukb-b-6458	2018	Mouth/teeth dental problems: Mouth ulcers	MRC-IEU	4, 61, 113	98, 51, 867

### Mendelian randomization analysis

In this study, we used inverse variance weighted (IVW) as the main analysis method. And the median weighted method, weighted mode method, MR Egger method, Simple mode method and Weighted mode method were used as Supplementary Method. The IVW principle used the reciprocal of the variance of each instrumental variable as a weight for weighted calculation under the premise of ensuring that all instrumental variables were effective, and the final result was the weighted average of the effect values of all instrumental variables. The above analysis was implemented through the TwoSampleMR package.

### Sensitivity analysis

**FIGURE 1:**
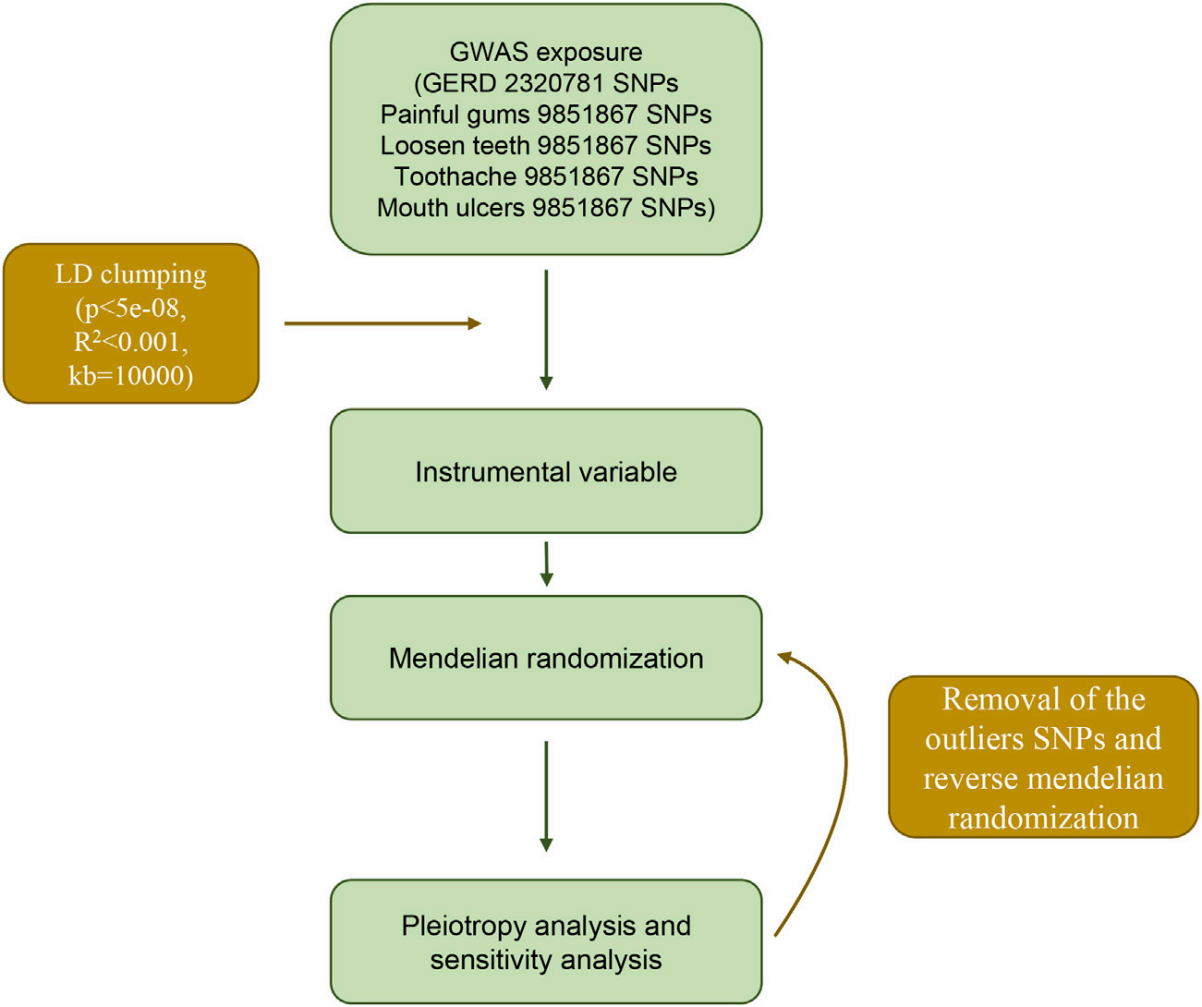
Flow chart and design of this mendelian randomization study.

First, we performed a heterogeneity test for the included instrumental SNPs using Cochran’s Q test and I^2^ statistic. If the test results suggested an existed heterogeneity, we further detected the outlier SNPs by MR-PRESSO package (Verbanck et al., 2018). And after removing the detected outliers SNPs, Mendelian randomization analysis was performed again and heterogeneity was checked again (Figure 1). The horizontal pleiotropy of instrumental variables was then detected by the MR-Egger method (Bowden et al., 2015). If the *p*-value of the intercept term of the regression equation is >0.05, it indicates that horizontal pleiotropy is not exhibited. Similarly, in order to verify the stability of the analysis results, we performed a leave-one-out analysis through the leave_one_plot () function in the TwoSampleMR package (Hemani et al., 2017). The funnel plot and forest plot were also generated by TwoSampleMR package. The principle of the leave-one-out method is to eliminate each SNP one by one and calculate the combined effect of the remaining SNPs, so as to determine whether the main effect of an instrumental variable leads to the causal relationship between the exposure factor and the outcome variable.

## Ethical approval

The GWAS summary data used in this study were obtained from published studies that have been approved by institutional review boards in their respective studies.

## Result

### Causal relationship between GERD and oral lesions

**FIGURE 2:**
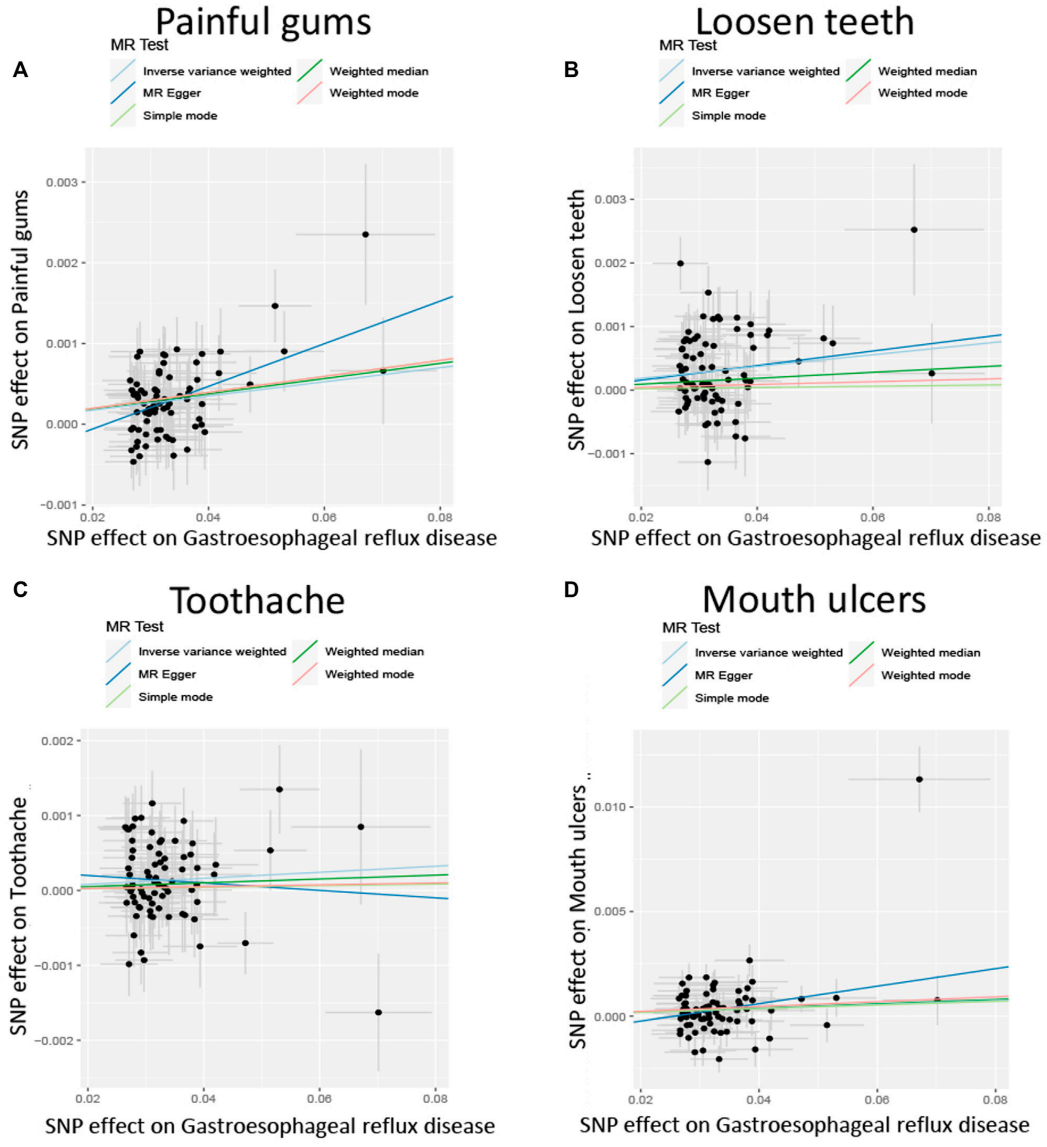
Scatterplot of the effect size for each SNP on GERD and the risk of **(A)** Painful gums, **(B)** Loosen teeth, **(C)** Toothache, **(D)** Mouth ulcers.

After screening the GERD GWAS summary statistics, a total of 80 SNPs were selected as instrumental variables (Supplementary Table S1). After mendelian randomization analysis using the TwoSampleMR package, IVW analysis showed that GERD showed a causal relationship with almost all oral lesions, including loosen teeth (*p* = 3.98E-06), oral ulcer (*p* = 0.00779079), bleeding gum (*p* = 0.01627596) and toothache (*p* = 0.01627596). *p* = 0.02197819). Also, MR Egger analysis, weighted median analysis and simple mode analysis also demonstrated a causal relationship between GERD and these common oral lesions (Table 2). In addition, although the slopes calculated by the different analyses were different, all analyses showed a positive relationship between GERD and oral lesions (Figure 2).

**TABLE 2:** MR analysis results of five common methods of GERD to painful gums, loosen teeth, toothache and mouth ulcers.

ID exposure	ID outcome	Outcome	Exposure	Method	nsnp	b	se	*p* val
ebi-a-GCST90000514	ukb-b-11161	Mouth/teeth dental problems: Painful gums || id:ukb-b-11161	Gastroesophageal reflux disease || id:ebi-a-GCST90000514	MR Egger	77	0.026394347	0.007459688	0.000695244
ebi-a-GCST90000514	ukb-b-11161	Mouth/teeth dental problems: Painful gums || id:ukb-b-11161	Gastroesophageal reflux disease || id:ebi-a-GCST90000514	Weighted median	77	0.009413891	0.001913832	8.70E-07
ebi-a-GCST90000514	ukb-b-11161	Mouth/teeth dental problems: Painful gums || id:ukb-b-11161	Gastroesophageal reflux disease || id:ebi-a-GCST90000514	Inverse variance weighted	77	0.008758933	0.001328755	4.34E-11
ebi-a-GCST90000514	ukb-b-11161	Mouth/teeth dental problems: Painful gums || id:ukb-b-11161	Gastroesophageal reflux disease || id:ebi-a-GCST90000514	Simple mode	77	0.009888257	0.004979183	0.050648794
ebi-a-GCST90000514	ukb-b-11161	Mouth/teeth dental problems: Painful gums || id:ukb-b-11161	Gastroesophageal reflux disease || id:ebi-a-GCST90000514	Weighted mode	77	0.009888257	0.004699471	0.038674976
ebi-a-GCST90000514	ukb-b-12849	Mouth/teeth dental problems: Loose teeth || id:ukb-b-12849	Gastroesophageal reflux disease || id:ebi-a-GCST90000514	MR Egger	77	0.011477439	0.011342248	0.31483016
ebi-a-GCST90000514	ukb-b-12849	Mouth/teeth dental problems: Loose teeth || id:ukb-b-12849	Gastroesophageal reflux disease || id:ebi-a-GCST90000514	Weighted median	77	0.004657994	0.002465903	0.058897043
ebi-a-GCST90000514	ukb-b-12849	Mouth/teeth dental problems: Loose teeth || id:ukb-b-12849	Gastroesophageal reflux disease || id:ebi-a-GCST90000514	Inverse variance weighted	77	0.009259929	0.002007526	3.98E-06
ebi-a-GCST90000514	ukb-b-12849	Mouth/teeth dental problems: Loose teeth || id:ukb-b-12849	Gastroesophageal reflux disease || id:ebi-a-GCST90000514	Simple mode	77	0.001000642	0.006571932	0.879385406
ebi-a-GCST90000514	ukb-b-12849	Mouth/teeth dental problems: Loose teeth || id:ukb-b-12849	Gastroesophageal reflux disease || id:ebi-a-GCST90000514	Weighted mode	77	0.002186752	0.006001517	0.716596105
ebi-a-GCST90000514	ukb-b-19191	Mouth/teeth dental problems: Toothache || id:ukb-b-19191	Gastroesophageal reflux disease || id:ebi-a-GCST90000514	MR Egger	77	-0.004975838	0.00990735	0.61697218
ebi-a-GCST90000514	ukb-b-19191	Mouth/teeth dental problems: Toothache || id:ukb-b-19191	Gastroesophageal reflux disease || id:ebi-a-GCST90000514	Weighted median	77	0.00254743	0.002373421	0.283129575
ebi-a-GCST90000514	ukb-b-19191	Mouth/teeth dental problems: Toothache || id:ukb-b-19191	Gastroesophageal reflux disease || id:ebi-a-GCST90000514	Inverse variance weighted	77	0.004038735	0.001763066	0.021978186
ebi-a-GCST90000514	ukb-b-19191	Mouth/teeth dental problems: Toothache || id:ukb-b-19191	Gastroesophageal reflux disease || id:ebi-a-GCST90000514	Simple mode	77	0.001047195	0.006625769	0.874837492
ebi-a-GCST90000514	ukb-b-19191	Mouth/teeth dental problems: Toothache || id:ukb-b-19191	Gastroesophageal reflux disease || id:ebi-a-GCST90000514	Weighted mode	77	0.001257552	0.006146903	0.838444763
ebi-a-GCST90000514	ukb-b-6458	Mouth/teeth dental problems: Mouth ulcers || id:ukb-b-6458	Gastroesophageal reflux disease || id:ebi-a-GCST90000514	MR Egger	77	0.04177879	0.019611096	0.036420579
ebi-a-GCST90000514	ukb-b-6458	Mouth/teeth dental problems: Mouth ulcers || id:ukb-b-6458	Gastroesophageal reflux disease || id:ebi-a-GCST90000514	Weighted median	77	0.009940645	0.003561053	0.005246591
ebi-a-GCST90000514	ukb-b-6458	Mouth/teeth dental problems: Mouth ulcers || id:ukb-b-6458	Gastroesophageal reflux disease || id:ebi-a-GCST90000514	Inverse variance weighted	77	0.009406138	0.003534807	0.007790791
ebi-a-GCST90000514	ukb-b-6458	Mouth/teeth dental problems: Mouth ulcers || id:ukb-b-6458	Gastroesophageal reflux disease || id:ebi-a-GCST90000514	Simple mode	77	0.008845566	0.008580587	0.305866062
ebi-a-GCST90000514	ukb-b-6458	Mouth/teeth dental problems: Mouth ulcers || id:ukb-b-6458	Gastroesophageal reflux disease || id:ebi-a-GCST90000514	Weighted mode	77	0.011547019	0.00807649	0.156899001

**FIGURE 3:**
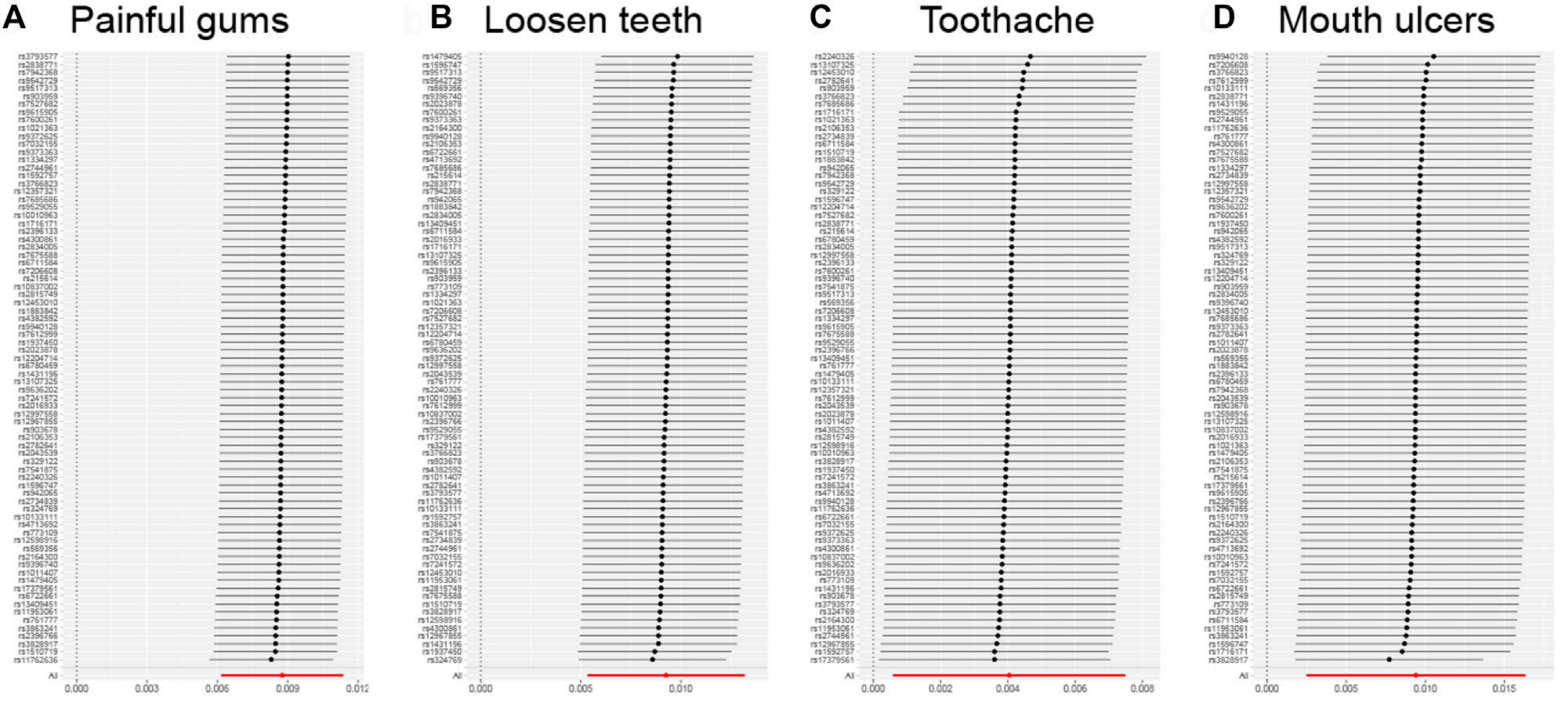
Leave-one-out analysis for the impact of individual SNPs on the association between GERD and the risk of oral lesions. **(A)** Painful gums, **(B)** Loosen teeth, **(C)** Toothache, **(D)** Mouth ulcers.

Stability analysis by leave-one-out method showed that no single SNP significantly altered the overall effect of GERD on several oral lesions (Figure 3), indicating the stability of our analysis. Also, the funnel plots and forest plots showed that there was no significant heterogeneity of the selected instrumental variable SNPs (Supplementary Figures S1, S2). We then performed a heterogeneity analysis and found a significant heterogeneity in the causal relationship between GERD and loosen tooth (MR Egger *p* = 0.000375318, IVW *p* = 0.000484792), bleeding gums (MR Egger *p* = 0.000140049, IVW *p* = 0.000163869), mouth ulcers (MR Egger *p* = 5.68E-08, IVW *p* = 1.62E-08). The heterogeneity between toothache and gum pain was not significant (Supplementary Table S2). After removing several detected outlier SNPs using MR-PRESSO analysis, heterogeneity analysis showed that all heterogeneity in the causal relationship was not significant between GERD and oral common diseases (Supplementary Tables S3, S4). We then performed the Mendelian randomization analysis and the results still suggested a causal relationship between GERD and oral lesions (Supplementary Table S3). Horizontal pleiotropy analysis showed that, except for gingival bleeding, there was no horizontal pleiotropy between several oral lesions and the occurrence of GERD (*p* > 0.05) (Supplementary Table S5).

## Discussion

In this study, we used a two-sample MR approach to analyze the causal relationship between GERD and oral lesions. We conclude that GERD is positively associated with an increased incidence of oral lesions in the European population. Our findings were valid and stable in IVW analysis before and after exclusion of outliers SNPs, and were also stable in sensitivity analysis.

Clinically, oral lesions are frequently observed in patients with GERD, where gastric contents (pH 1–1.5) consisting of acids, pepsin, bile salts, and trypsin may reflux to the esophagus and reach the oral cavity, leading to high levels of dental erosion and sometimes caries, and may also cause damage to oral soft tissues that are not adapted to their harmful potential (Tjon et al., 2021; Ribolsi et al., 2022). Various studies have demonstrated an increased prevalence of tooth erosion and caries in individuals with GERD compared to controls. At present, there are the following reports and speculations about the causes of different oral lesions caused by GERD.

Dental erosion refers to the reduction of dental mineralization due to chemical or ionization processes caused by non-bacterial factors (Donovan et al., 2021), and is not associated with bacterial infection. Hydroxyapatite crystals in tooth enamel can disintegrate in an acid environment with pH lower than 5.5, while the pH of refluxed gastric contents is usually lower than 2.0, which is conducive to the occurrence of tooth erosion (Shellis et al., 2014). Following tooth erosion, incomplete tooth surfaces are more susceptible to friction and wear, resulting in occlusal wear and loss. In addition, GERD patients often have abnormal esophageal motility, which is closely related to delayed acid clearance. Usually under physiological conditions, gastric reflux is caused by swallowing to induce peristaltic return to the stomach or by stimulating the esophageal mucosa to induce secondary peristaltic clearance. In GERD patients, however, this process is often impeded, and therefore acid clearance is delayed. A study of esophageal motility in patients with tooth erosion found a mean of 8% in patients with tooth erosion and 0% in healthy controls, suggesting that poor esophageal motility may be a risk factor for tooth erosion (Bartlett et al., 2000). Similarly, acidity from gastroesophageal reflux can also lead to oral soft tissue lesions (Watanabe et al., 2017). In GERD patients, palatal mucosal epithelial atrophy and increased fibroblasts were observed. However, these changes which are only detected by morphometry (Silva et al., 2001). In another study, soft/hard palate and uvula erythema and a burning sensation in the mouth were more common in GERD patients (Di Fede et al., 2008). GERD has been reported to cause esophageal mucosal damage and esophagitis (Mari et al., 2022). But so far, there have been very limited reports on whether GERD can lead to oral ulcers. In an experimental model of rat chronic acid reflux esophagitis, in addition to tooth erosion, the researchers also observed inflammatory cell infiltration in the mucosa of the back of the tongue, proving that acid reflux can also lead to an inflammatory response in the oral mucosa (Shimazu et al., 2018). Our study is the first to identify a causal relationship between GERD and oral ulcers through genetic evidence.

The effect on the secretion and properties of saliva is also one of the important causes of oral lesions caused by GERD. Under physiological conditions, the removal of acidic substances in esophageal reflux includes peristaltic clearance and salivary chemical clearance. Saliva not only buffers acid, but also stimulates esophageal motility after being swallowed, causing further acid removal. Therefore, saliva is considered to be an important protective mechanism of the esophagus and oral mucosa against acid reflux, and both the quality and quantity of saliva secretion directly affect the occurrence of dental erosion. In addition to its role in buffering the acidic environment, saliva also plays a major role in the maintenance of oral health and the repair of hard and soft oral tissues, including antibacterial effects, promotion of remineralization and wound healing. A reduction in saliva flow can speed up the process of tooth erosion. Likewise, a reduction in saliva, along with changes in its quality, is thought to be the main cause of periodontitis in GERD patients. In the study of Song et al. (2014), GERD was considered as an independent risk factor for periodontitis.

In addition, considering that proton pump inhibitors are currently recommended as the preferred treatment for GERD, the use of proton pump inhibitors (PPI) is thought to affect the secretion and properties of saliva. In GERD patients taking PPIs, their salivary flow rate was significantly lower than controls, and their acid buffering capacity decreased (Tanabe et al., 2021). Therefore, drug use accompanying GERD may also be a causative factor for oral lesions.

Despite the validity and robustness of our MR results, the current study has some limitations. First, since the GWAS data for oral lesions and GERD used in this study were derived from European populations, our findings may not be scalable to other populations. Second, since large-scale oral lesion GWAS are rarely published, we selected only the phenotypes of the major oral lesions disclosed in the UKBB. However, most of these oral lesion phenotypes are local manifestations of common oral diseases (caries, periodontitis) rather than directly representing the occurrence of these diseases. The causal relationship between these diseases and GERD remains to be further confirmed. Finally, the occurrence of all these oral lesions is determined by a combination of genetic as well as environmental factors, and our results only partially explain the causal effect of GERD on oral lesions.

In conclusion, our study demonstrated a causal relationship between GRED and several common oral lesions, including toothache, loose teeth, bleeding gums, painful gums, and oral ulcers, through mendelian randomization analysis. When the MR analysis was repeated after removing outlier SNPs, our results were still robust. The present findings suggest that interventions should be taken to prevent the occurrence of oral lesions in patients with confirmed GERD. Considering that a large number of undiagnosed GERD patients showed oral lesions as the first symptom, dentists should consider GERD in the etiological analysis of these common oral lesions, especially tooth erosion.

## Data Availability

The original contributions presented in the study are included in the article/Supplementary Material, further inquiries can be directed to the corresponding author.

## Supplementary material

SUPPLEMENTARY TABLE S1 The instrumental variation SNPs selected for MR analysis.

SUPPLEMENTARY TABLE S2 The heterogeneity results of MR analysis from the Cochran’s Q test.

SUPPLEMENTARY TABLE S3 The instrumental variation SNPs selected for MR analysis after removal of outlier SNPs.

SUPPLEMENTARY TABLE S4 The heterogeneity results of MR analysis from the Cochran’s Q test after removal of outlier SNPs.

SUPPLEMENTARY TABLE S5 The pleiotropy analysis results of MR analysis.
