# Supplementary figures and images for: Exploring the causal relationship between gastroesophageal reflux and oral lesions: A mendelian randomization study
Source: Front Genet. 2022 Nov 29;13:1046989. doi: 10.3389/fgene.2022.1046989 (PMC9745088; doi:10.3389/fgene.2022.1046989)

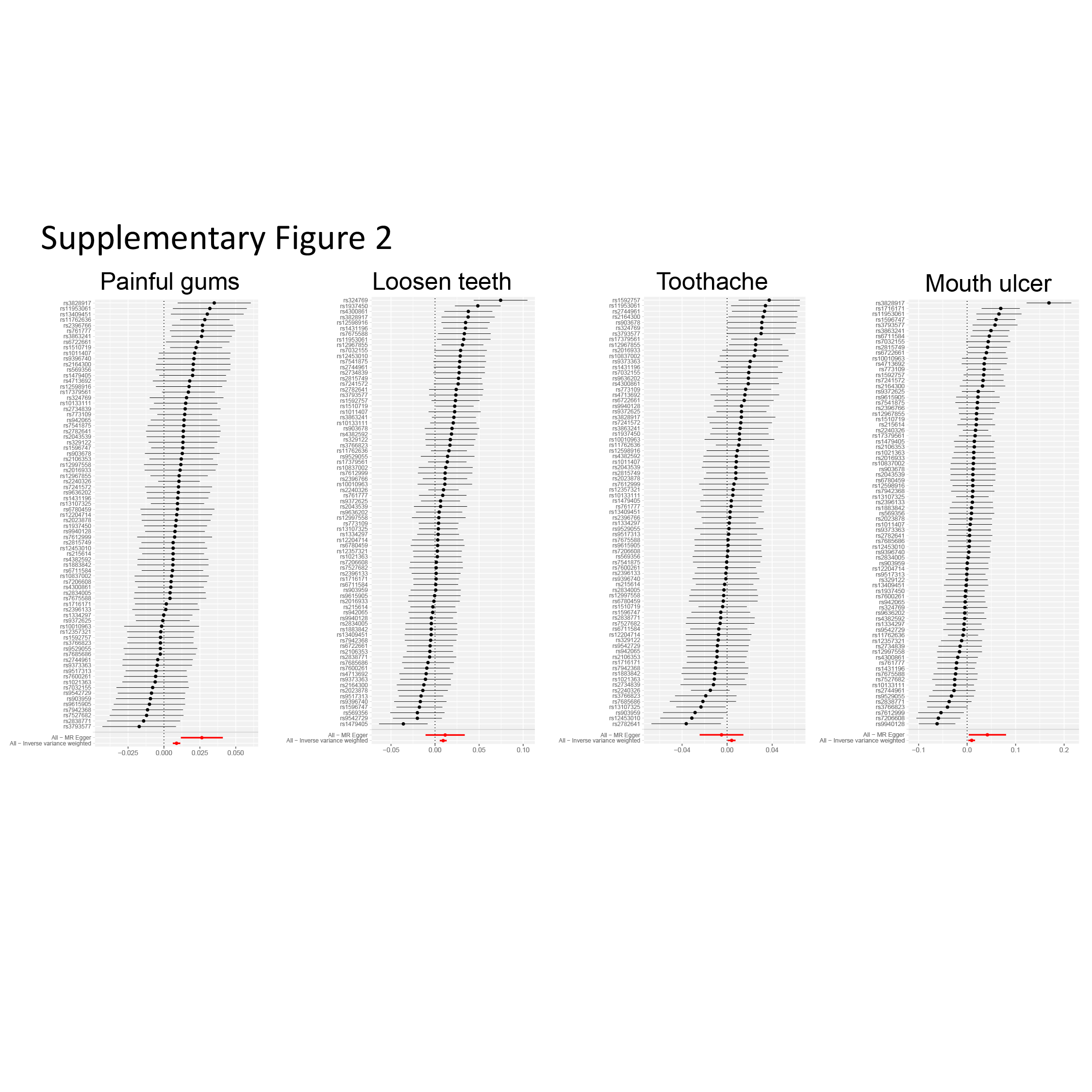

Supplement: Supplementary file 1 [file Image2.jpg]

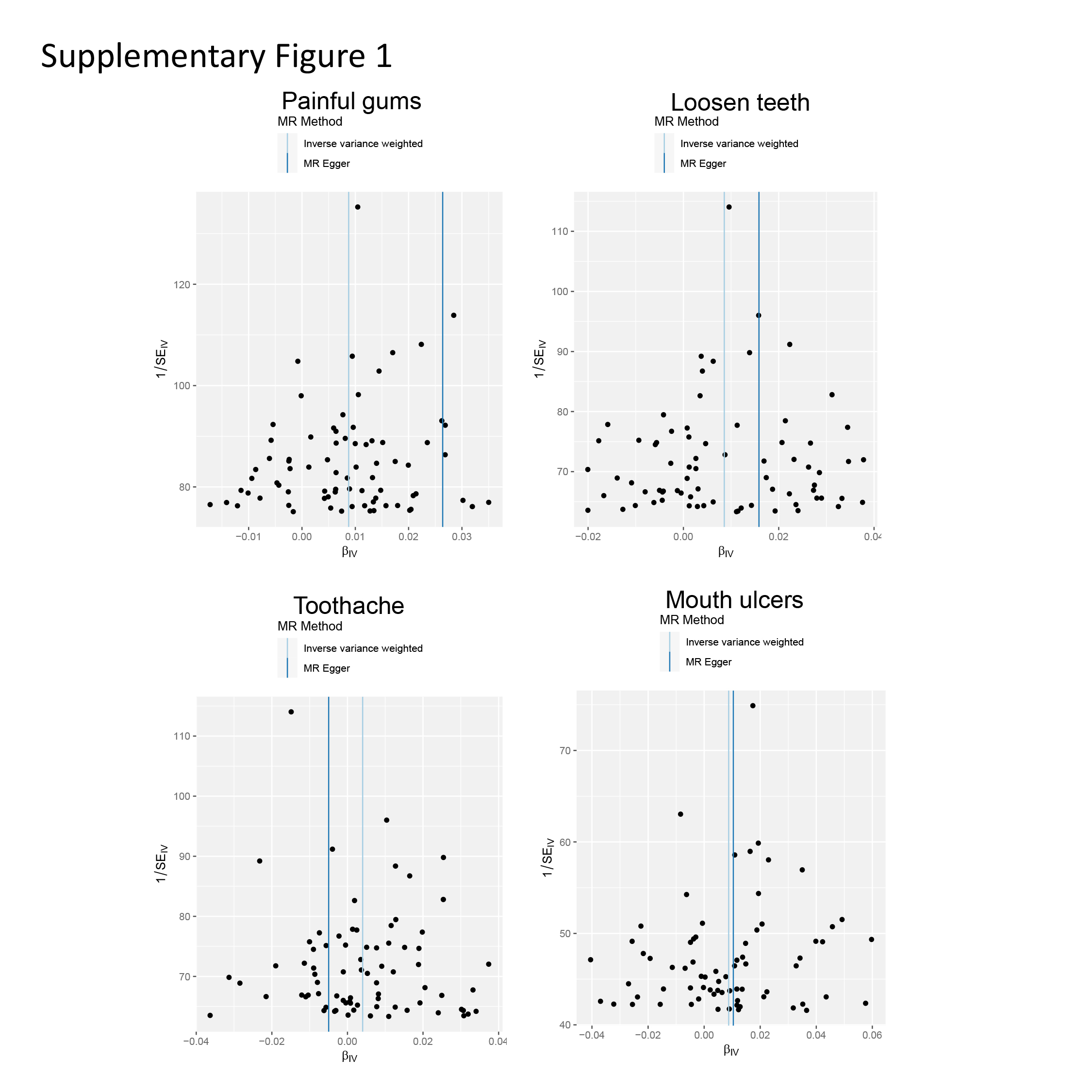

Supplement: Supplementary file 5 [file Image1.jpeg]
